# Immediate and delayed signal of slab breakoff in Oligo/Miocene Molasse deposits from the European Alps

**DOI:** 10.1038/srep31010

**Published:** 2016-08-11

**Authors:** Fritz Schlunegger, Sébastien Castelltort

**Affiliations:** 1 Institute of Geological Sciences, University of Bern, Baltzerstrasse 1+3, CH-3012 Bern, Switzerland; 2 Department of Earth Sciences, University of Geneva, Rue des Maraîchers 13, 1205 Geneva, Switzerland

## Abstract

High-resolution 32–20 Ma-old stratigraphic records from the Molasse foreland basin situated north of the Alps, and Gonfolite Lombarda conglomerates deposited on the southern Alpine margin, document two consecutive sedimentary responses - an immediate and delayed response - to slab breakoff beneath the central Alps c. 32–30 Ma ago. The first signal, which occurred due to rebound and surface uplift in the Alps, was a regional and simultaneous switch from basin underfill to overfill at 30 Ma paired with shifts to coarse-grained depositional environments in the foreland basin. The second signal, however, arrived several million years after slab breakoff and was marked by larger contributions of crystalline clasts in the conglomerates, larger clast sizes, larger sediment fluxes and shifts to more proximal facies. We propose that this secondary pulse reflects a delayed whiplash-type erosional response to surface uplift, where erosion and sediment flux became amplified through positive feedbacks once larger erosional thresholds of crystalline bedrock were exceeded.

Progradation of coarse-grained material in foreland basins has been related to tectonic uplift, which accentuates erosion through the generation of steeper slopes^1^, or to shifts towards stormier climates, which enable the transport of larger clasts by more powerful floods^2^,^3^. Most of these interpretations assume instantaneous process-responses, but recent physical models suggest that sediment supply signals linked with external perturbations can be buffered or even amplified^1^,^4^,^5^, with a possible time lag^6^. Despite this progress, interpretations of depocenter progradation have remained non-unique mainly due to a lack of independent chronologies for the driving force in the hinterland where the sediment sources are, and the stratigraphic response in the adjacent sedimentary basin. Here, we approach this problem taking advantage of well dated^7^,^8^,^9^ 32–20 Ma-old sedimentary archives encountered at three sections within the Molasse foreland basin (Fig. 1a), and geochronological constraints from the adjacent European Alps^10^,^11^,^12^,^13^,^14^.

The Central European Alps (Fig. 1a) comprise a doubly-vergent nappe stack with a crystalline core of European origin exposed in the Lepontine dome (L on Fig. 1a) that straddles the subducting European plate^14^. The present-day architecture of the orogen is the consequence of a subduction-collision history, which started with the subduction of the European oceanic lithosphere beneath the Adriatic continental plate and the closure of the Tethys Ocean during the Late Cretaceous^14^. At c. 35 Ma, the European continental lithosphere entered the subduction channel, where the contrasts in flexural rigidities between the subducted oceanic lithosphere and the continental European plate induced extensional stresses within the slab, with the result that the oceanic lithosphere slab broke off 30–32 Ma ago^10^,^11^,^12^,^13^,^14^ (Fig. 1b). Slab delamination was associated with the ascent of magmas to shallow crustal levels (e.g., Bergell intrusion labeled as B in Fig. 1a)^10^,^11^,^12^,^13^,^14^, rapid rock uplift and orogen-parallel extension in the rear of the Alps. Uplift and extension was accomplished through backthrusting along the Insubric Line (IL on Fig. 1b) and orogen-parallel slip along low-angle detachment faults^15^,^16^,^17^. Backthrusting and related rock uplift resulted in the rise of the Alpine topography^18^, which in turn caused an increase in sediment flux^19^ to the adjacent sedimentary basins. The rise of the Alpine topography continued until c. 25–20 Ma, when the mountain belt reached a cross-sectional width of c. 150 km and a total relief (i.e., elevation difference between the foreland basin and the major fluvial drainage divides in the Alps) of c. 1500–2500 m^18^ that has been maintained until today^18^.

The Molasse foreland basin, situated on the northern side of the Alps^19^ (Fig. 1a), hosts the erosional detritus of the evolving orogen^7^,^19^,^20^,^21^,^22^,^23^. Surface uplift in the back of the Alps after removal of the oceanic lithosphere 32–30 Ma ago (Fig. 1b) and the related increase in sediment discharge to the basin^19^ (Fig. 2a) was linked with the change from ‘flysch’-type underfilled sedimentation prior to 30 Ma, to terrestrial and shallow marine ‘molasse’-type conditions thereafter^20^,^21^ (Fig. 2b). Large sediment fluxes also controlled the build-up of alluvial megafans^22^ (Fig. 2b,c). These systems evolved through coalescence, when several smaller fans a few kilometers wide merged to three major depocentres in the Swiss part of the basin (Napf, Rigi, Hörnli) between 30 and 25 Ma, and finally to two major megafans after 22 Ma (Napf, Hörnli)^18^,^22^. The fans expanded radially into the foreland basin over several tens of kilometers, where they either merged with an axial fluvial system (Fig. 2c,d), or with a shallow peripheral strait that linked the Tethys with the Black Sea^20^,^22^ (shallow marine deposits on Fig. 2b). Close to the apex adjacent to the Alpine thrust front, the megafans were laterally encroached by locally-derived ‘bajada’ fans (Fig. 2c,d) with sources in the frontal Alpine nappes^24^. The material in the basin was coarsest at the fan apex, from where it became finer grained towards more distal sites^7^,^24^. These proximal-distal trends were also associated by a decrease in the cumulative thicknesses of deposited material towards the distal basin margin (Fig. 2c), with the effect that the basin adopted a wedge-shaped cross-sectional geometry (Fig. 2d)^24^.

Between approximately 30 and 20 Ma, the rivers feeding the Napf, Rigi and Hörnli megafans were the largest dispersal systems with sources in the central Alps. These streams captured a large portion of their clastic material from sedimentary and crystalline thrust nappes that were overlying the Lepontine dome at that time (Fig. 1a)^7^,^23^,^24^,^25^,^26^. In the Molasse basin, related sedimentary archives are well exposed in three sections (Fig. 1a), situated at the proximal basin border next to the megafan depocenters. On the southern side of the Central Alps, Late Oligocene to Early Miocene submarine coarse-grained debris flows deposits of the Gonfolite Lombarda group (G on Fig. 1a) also record the response to the rise of the orogen and related changes in surface erosion^14^.

Here, we use the archives in the Molasse basin to document two consecutive sedimentary responses - an immediate first and a delayed second signal - to slab breakoff beneath the central Alps. The first signal was characterized by a shift from the underfilled ‘flysch’-type to the overfilled ‘molasse’-type of basin evolution^20^,^21^. We show that several millions of years later, the arrival of the second signal was marked by: distinct pulses in sediment discharge paired with the supply of material with larger grain sizes and larger relative abundance of crystalline clasts derived from the back of the Alps. We propose that these sediment pulses reflect whiplash-type^27^ erosional responses to slab breakoff, where erosion of Alpine streams became amplified once the larger erosional thresholds of the crystalline bedrock were exceeded. This mechanism, which has been applied in sediment flux-dependent incision models^27^, describes the whip-like upstream propagation of an erosional wave following an increase in rock uplift rate. In the present case study, the availability of well-constrained chronological data from the hinterland and the adjacent sedimentary basin allows us to document for the first-time the arrival of two consecutive signals in response to a single driver in a natural setting. Our results also indicate that the landscape’s response to a deep-seated tectonic event can take several millions of years. This implies that related responses in sedimentary basins to such events can be significantly protracted (several Ma) and possibly non-unique.

## Results

### Western Molasse basin– the Napf megafan

**Figure 1:**
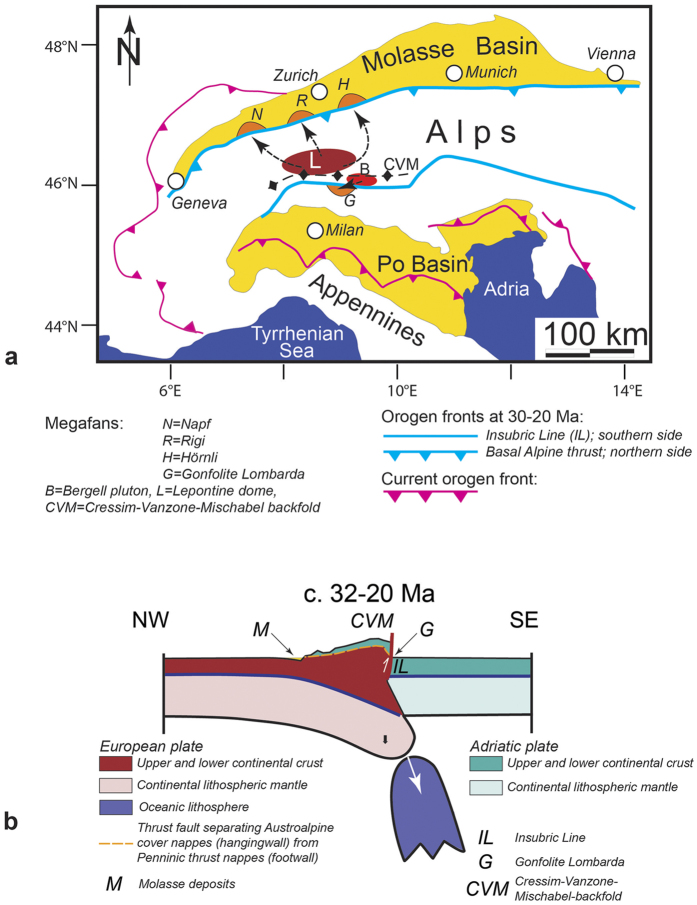
The Alps. (**a)** Geologic map illustrating locations of megafans together with areas in the Alps where the material sources have been located between the Late Oligocene and the Early Miocene. L = Lepontine dome situated in the back of the Alps that was considered to represent the area where the crystal detritus was derived from; B = Bergell pluton; CVM = Cressim-Vanzone-Mischabel backfold; IL = Insubric Line. The map has been drawn based on Schlunegger and Kissling^18^ using Illustrator 15.1.0 licenced to Uni Bern. (**b**) Restored section of the Alps for the Late Oligocene^18^ together with the locations where the megafans were deposited. Slab breakoff beneath the back of the Alps caused a rebound of the European lithosphere, which was accomplished by backthrusting along the Insubric Line (IL) together with the build of the Alpine topography. The Cressim-Vanzone-Mischabel (CVM) backfold was the major drainage divide between the Late Oligocene and the Early Miocene^14^,^15^. The section runs from Zurich to Milan (Fig. 1a). The section has been drawn based on Schlunegger and Kissling^18^ using Illustrator 15.1.0 licenced to Uni Bern.

**Figure 2:**
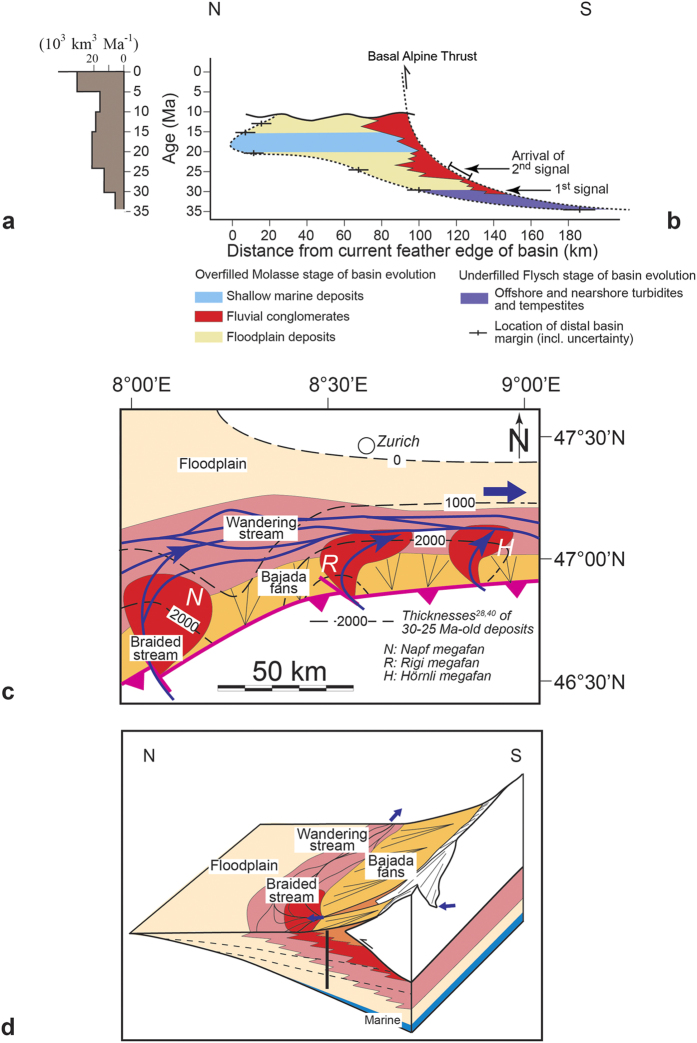
The Molasse basin. (**a**) Sediment discharge from the Central Alps to the Molasse basin^19^, which has been measured based on preserved volumes of rocks derived through erosion of the Alps (figure adapted from Schlunegger and Kissling^18^). (**b**) Stratigraphic architecture of the Molasse deposits within a temporal framework^18^. Note that the change from basin underfill to overfill c. 30 Ma ago (1^st^ signal) coincides with the time when the topography started to build up in response to slab breakoff (Fig. 1b). The arrival of the 2^nd^ signal was marked by rapid fan progradation (based on Schlunegger and Kissling^18^). (**c**) Restored palaeogeographic situation of the Molasse basin at c. 25 Ma^28^,^40^. The dashed lines indicate the cumulative thicknesses of material that accumulated between 30–25 Ma^28^,^40^. The map has been drawn on the basis of Pfiffner *et al*.^40^ using Illustrator 15.1.0 licenced to Uni Bern. (**d**) 3D reconstruction of the architecture of the Molasse basin at c. 25 Ma based on Schlunegger *et al*.^24^. The floodplain represents an alternation of sandstones and mudstones; wandering streams accumulated a succession of alternated conglomerate and mudstone beds; braided streams are recorded by amalgamated conglomerate beds. Local fans, or ‘bajada’ fans, are recognized by alternated debris-flow conglomerates and mudstones (see also Fig. 3). The dashed lines represent isochrones. The sections of Fig. 3 (black vertical line) are slightly offset of the fan apex.

**Figure 3:**
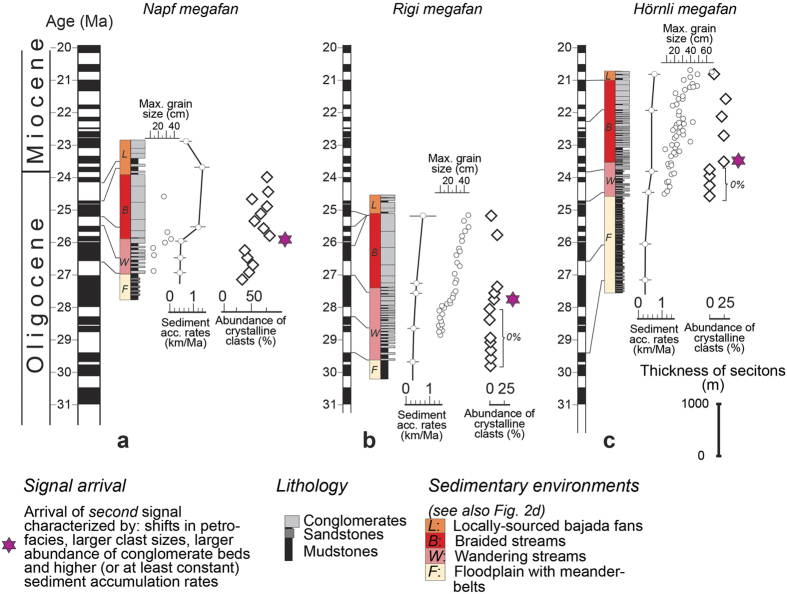
Stratigraphic data from the Molasse basin. (**a)** Stratigraphic development of the Napf megafan together with magnetostratigraphic calibration of the section^8^, accumulation rates of non-decompacted sediments, evolution of maximum clast sizes^28^ and evolution of relative abundance of crystalline clasts^29^. This section records the progradation of the Napf megafan (Fig. 1a). (**b**) Data from the Rigi fan, showing magnetostratigraphic calibration of the section^9^, sediment accumulation rates of non-decompacted material, development of maximum clast sizes plus first arrival of crystalline clasts^25^. (**c)** Data from the Hörnli megafan, including: stratigraphic architecture and chronological calibration of the section^7^, evolution of largest clasts^30^, sediment accumulation rates^7^ and relative abundance of crystalline clasts in the Hörnli megafan deposits^26^,^30^ (Fig. 1a). See Fig. 2d for palaeogeographic sketch and color codes for environments.

The Napf megafan conglomerates, situated at c. 100 km distance from the site of slab delamination (Fig. 1a), are the westernmost sedimentary deposits that we use here to infer the arrival of a distinct erosional signal. A 3500 m-thick section situated at the proximal basin border at 46°47′N and 7°43′E exposes a suite of alternated marls, sandstones and conglomerates (Fig. 3a) that were deposited c. 30–24 Ma ago^8^. The lowermost quarter of the section, dated to c. 28–26.5 Ma^8^, comprises an alternation of c. 5 m-thick cross-bedded river-belt sandstone beds and several m-thick mudstone interbeds with root casts and a mottling fabric. This succession has been assigned to a meander-belt environment with laterally extended floodplains (Fig. 2d, and F on Fig. 3a)^28^. This succession is overlain by a suite of up to 5 m-thick massive- and cross-bedded conglomerate beds with interbedded mudstones containing caliche nodules and root casts. These conglomerates host clasts derived from the entire Alps and record the supply of sediment by streams with wandering channels (unit labeled with W on Fig. 3a)^28^. From 25.5 Ma onwards, the conglomerates coarsen and thicken upwards towards a succession where amalgamated conglomerate beds with a massive fabric dominate the stratigraphic architecture. This change records a transition to a braided river system (unit labeled as B on Fig. 3a) where overbank fines, represented by red-mottled mudstone interbeds, are rare^28^. Clast imbricates are more frequent up-section, and the largest clasts increase from <15 cm prior to 25.5 Ma to >30 cm thereafter^28^.

The 25.5 Ma-old shift from alternated conglomerates and mudstones to the suite of amalgamated conglomerate beds with outsized clasts up to 30 cm in diameter represents a remarkable break in the stratigraphic architecture (red star; Fig. 3a), mainly because it is also associated with a shift in petrofacies. In particular, the relative abundance of crystalline clasts increased from <50% prior to 25.5 Ma to >70% thereafter, and epidote crystals started to dominate the heavy mineral composition by >80% (ref. 29). Mapping reveals that this shift in litho- and petrofacies can be traced over tens of kilometers across the basin^29^. Also at 25.5 Ma, sediment accumulation rates, estimated through thicknesses of non-decompacted deposits, increased from 0.4 mm/yr to >1 mm/yr (Fig. 3a)^28^.

The sedimentary architecture and composition of the top member of the section (c. 500 m thick, Fig. 3a) differs from the conglomerates below. Up to 5-m thick, deeply scoured conglomerate beds, sometimes matrix-supported, display a ribbon geometry and a monomict composition where flysch clasts are the major constituents^28^,^29^. This unit has been interpreted as recording the supply of material from the Alpine frontal nappes to bajada fans (unit labeled as L on Fig. 3a) through torrential floods and debris flows^28^.

### Central Molasse basin – the Rigi megafan

The Rigi megafan chronicles the arrival of the inferred erosional signal at c. 80 km distance from the back of the Alps (Fig. 1a). This megafan has a cross-sectional width of c. 20–30 km and hosts Late Oligocene fluvial deposits^24^.

A well-exposed section is encountered at the proximal margin of the basin at 47°04′N and 8°29′E (Fig. 3b). The entire section is c. 3500 m thick and comprises a suite of mudstones, sandstones and conglomerates^25^, which was dated to the time interval between c. 30 and 26–25 Ma through magneto-polarity chronologies^9^. Similar to the Napf conglomerates, the lowermost three quarters of the Rigi section, c. 3000 m thick, expose a large-scale coarsening and thickening upward megasequence^25^. It starts with alternating mudstone and massive- to cross-bedded sandstone beds typical for a meander belt environment that was bordered by a broad floodplain. This sandstone-mudstone alternation is overlain by a suite of conglomerates and mudstones where thicknesses of individual conglomerate beds range between 5 and 10 m. A marked change occurred at 27 Ma (red star on Fig. 3b) when sedimentation shifted to an amalgamated stack of massive-bedded conglomerate beds where red-mottled mudstone interbeds are rare^24^. Similar to the Napf deposits, this shift in the sedimentary architecture reflects a major change in the dispersion pattern where sediment deposition by wandering streams gave way to sediment dispersal by braided streams on an alluvial megafan^24^. However, this shift in sedimentation occurred 1.5 Ma earlier than at Napf (Fig. 3). The stratigraphic change at Rigi was also associated with the first arrival of red granite clasts^25^. Provenance tracing revealed that these clast types were derived from the crystalline basement of the Austroalpine nappes^25^ that were exposed in the back of the Alps during the Late Oligocene. Also at that time, the maximum clast sizes increased from <15 cm to >30 cm, and sediment accumulation rates increased from <0.4 mm/yr to >0.6 mm/yr, or remained constant, depending on how the uppermost magnetozone at Rigi is correlated with the magneto-polarity time scale (MPTS, Fig. 3b)^9^.

The deposits of the uppermost c. 500 m of the section (Fig. 3b) are characterized by alternating mudstones and 3–5 m-thick conglomerate beds^25^. The conglomerates host angular to subrounded clasts with a monomict composition where flysch clasts are the dominant constituents^25^. These sedimentary characteristics were considered to point to the occurrence of debris flows and torrential floods with local sources situated at the orogen front^24^.

### Eastern Molasse basin – the Hörnli megafan

The Hörnli megafan, situated at c. 120 km from the back of the Alps (Fig. 1a), is the farthest system that records the arrival of the here inferred erosional signal. An approximately 4000 m-thick section, dated to c. 30–20 Ma according to magneto-polarity stratigraphies (Fig. 3c), is encountered at 47°16′N and 9°13′E adjacent to the Alpine front. Similar to Napf and Rigi, the section displays a large-scale coarsening- and thickening upward megasequence deposited by perennial streams with sources in the central Alps east of the Lepontine dome^7^. The section begins with alternating sandstone-mudstone beds and evolves into a conglomerate-mudstone succession. The dispersal pattern experienced a distinct change at 23.8 Ma (red star on Fig. 3c), when an amalgamated stack of conglomerate beds started to dominate the stratigraphic architecture^7^. Similar to Napf and Rigi, this change in the sedimentation pattern reflects a shift towards a braided stream on an alluvial megafan^7^. Also similar to Rigi and Napf, this shift in deposition occurred contemporaneously with a marked change in petrofacies, which is characterized at Hörnli by the first arrival of crystalline material^7^. These clast types were derived from the basement nappes of the Austroalpine domain^26^ that were exposed east of the Lepontine dome during that time (Fig. 1a). The 23.8 Ma-old change in sedimentation pattern was also associated with an increase in the size of the largest clasts from <15 cm to >20–30 cm^7^,^30^, and with a shift towards higher sediment accumulation rate from originally <0.3 mm/yr prior to 23.8 Ma, to >0.3 mm/yr thereafter^7^. Accordingly, while the Hörnli deposits chronicle the same shifts in sedimentary dynamics as the strata at Napf and Rigi, the abrupt change in the sedimentation pattern and petrographic composition occurred c. 3.2 Ma later than at Rigi, and 1.7 Ma later than at Napf (Fig. 3).

### Stratigraphic data from the Southern side of the Alps

On the southern side of the Alps, at c. 15 km distance from the locus of slab breakoff, the Late Oligocene Gonfolite Lombarda deposits (Fig. 1a) are made up of an amalgamated stack of matrix-supported conglomerates with outsized clasts, which has been interpreted to reflect the deposition by debris flows supplied within a submarine canyon^31^. Embedded granitic clasts that were derived from the Bergell pluton c. 10–20 km farther north^32^ (labeled as B on Fig. 1a) imply that the cover rocks of the Alpine pluton had already been removed at that time.

## Discussion

**Figure 4:**
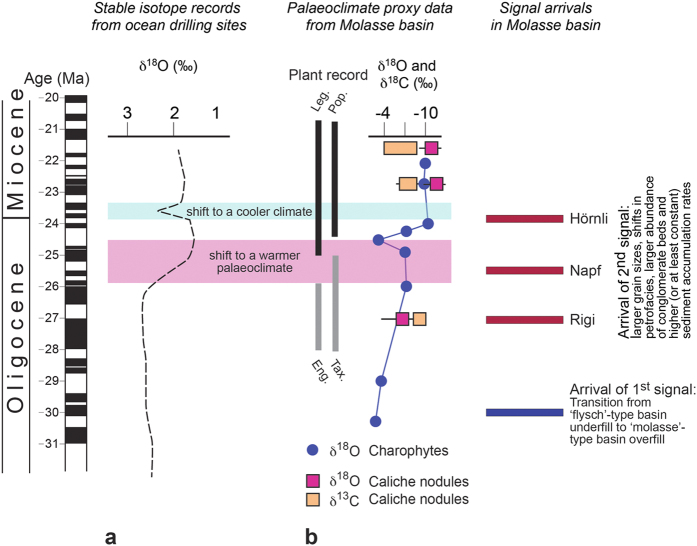
Palaeoclimate proxy data. (**a**) Evolution of palaeoclimate based on δ^18^O records^36^. (**b)** Paleoclimate proxy data collected in the Molasse Basin^37^,^38^,^39^. Leg. = Leguminosae, Pop. = Populoid, Eng. = Engelhardia, Tax. = Taxodiacea.

The three Molasse sections described here are all characterized by a similar, yet diachronous, abrupt sedimentation change (red stars on Fig. 3) within an overall coarsening and thickening-upward trend. Such a shift in the stratal architecture could be interpreted as the stratigraphic response to local tectonic events in the immediate hinterland of each fan^7^,^9^,^20^,^28^,^33^, driven by the northward shift of the Alpine orogen (e.g., Fig. 2d)^34^. Alternatively, these shifts in sedimentation patterns could also have occurred in response to a transition towards more erosive climates, yielding larger sediment fluxes^2^ and causing the megafans to prograde into the basin^4^,^35^. Here, we explain why we discard both of these possibilities. First, while thrusting at the range front (Fig. 2d) coupled with northward progradation of the Alpine edifice could explain that each section terminates with debris flows deposits and bajada fans with sources situated in the Alpine border (Fig. 3), these mechanisms alone are not capable of explaining the arrival of a substantial proportion of crystalline clasts in the middle of the sections (red stars on Fig. 3). Such distinct petrographic changes, paired with shifts towards amalgamations of coarse-grained conglomerate beds, invoke increased erosion in the back of the Alps (Fig. 1b) rather than at the Alpine front. Second, models predict that higher sediment accumulation rates (Fig. 3) would be associated by fining-upward rather than coarsening-upward trends as documented here, if local tectonics alone would be the major driving force^20^,^33^. Accordingly, we do not consider tectonic processes at the Alpine front alone as a viable mechanism to explain the observed changes in the Molasse stratigraphies. In the same sense, we discard the possibility that the Molasse trends could have occurred in response to shifts towards more erosive climates. Palaeoclimate proxy data are available from global deep-sea oxygen and carbon isotope records that were compiled from more than 40 drilling sites around the globe (Fig. 4a)^36^. Most of the data were collected from the long-lived benthic taxa *Cibicidoides* and *Nuttallides* that are embedded in pelagic carbonate-rich mud^36^. At a more local scale, information about palaeoclimate conditions during deposition of the Molasse units (Fig. 4b) are based on the fossiliferous records of plants embedded in overbank fines^37^,^38^, and carbon and oxygen isotope values recorded by caliche nodules in Molasse palaeosoils^39^ and charophytes^38^. Although both global stable isotope records^36^ and local proxy datasets^37^,^38^,^39^ do suggest that climate conditions did change between the Late Oligocene and the Middle Miocene (Fig. 4), it is unlikely that a climate driver alone is capable of explaining the recorded changes within the Molasse sections. We base this inference on the observation that identical shifts in the depositional architecture and provenance records occurred under a cooling (Hörnli) or warming (Napf) palaeoclimate, or are not related to any palaeoclimate shifts (Rigi) (Fig. 4). In summary, neither local thrusting along the Alpine thrust front nor global and local climate changes alone are capable to explain the here reported changes in the stratigraphic architectures. Instead, we propose a scenario where slab delamination at lithospheric levels beneath the back of the Alps and related surface uplift (Fig. 1b) explain the changes observed in the sedimentation patterns. These mechanisms are outlined in the next paragraph.

In this context, we first recall that the three sections, situated at different locations, chronicle the same observations as they record the arrival, at different times, of a unique signal characterized by: larger grain sizes, larger contributions of crystalline constituents, more frequent occurrence of braid plain conglomerates beds, and constant or increasing sediment accumulation rates (signal arrival marked by red stars on Fig. 3). Conceptual models suggest that such a change can be diagnostic of an augmentation in sediment supply to the basin, rather than an increase in water discharge or a decrease in subsidence^1^. In addition, the changes towards predominance, or first appearance, of crystalline material (Hörnli, Rigi) or greenshist quartzite clasts, with abundant epidote crystals in the heavy mineral suites (Napf) suggest a shift in the site of erosion. Paleogeographic restorations of tectonic shortening imply that related lithologies were exposed in the crystalline thrust nappes that were located in the back of the Alps surrounding the Lepontine dome (labeled L on Fig. 1a)^14^,^24^. These provenance constraints imply that the change in the stratigraphic architecture towards a suite of amalgamated conglomerates was linked with a significant increase in surface erosion rates and related exhumation in the back of the Alps^24^,^25^,^26^,^29^.

**Figure 5:**
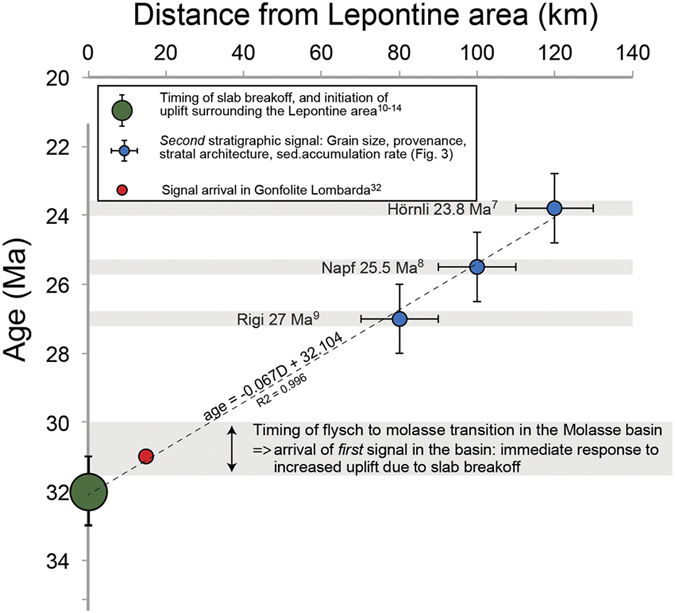
Propagation of erosional signal through space and time. Diagram illustrating the dependency of the arrival time of the inferred signal as a function of the cross-sectional distance between the back of the Alps (Lepontine area, Fig. 1a) and the space of entry into the sedimentary basins. Since this distance was largest for the Hörnli deposits (Fig. 1a), the signal arrived with the largest delay. The first signal, characterized by the change from basin underfill to overfill, was nearly contemporaneous with slab breakoff and marks the immediate growth of the topography.

Here, we present chronological evidence from the foreland basin deposits to further support the statement that slab breakoff was the major driving mechanism for the changes in erosion and shifts in megafan deposition. To this extent, we constrain the timing of the perturbation responsible for the observed stratigraphic response based on the following: Let us consider the restored distances of 120 km, 100 km, 80 km for the lengths of the streams between the Late Oligocene drainage divide situated in the back of the Alps (Lepontine dome), and the apexes of the Napf (100 km), Rigi (80 km) and Hörnli (120 km) megafans^14^,^40^, and let us take into account ±10 km of uncertainty on the location of each section. A regression analysis of the signal arrival time as a function of distance from the back of the Alps yields 29.43–33.43 Ma (Fig. 5) for the intercept at origin (R2 = 0.99). This age should then represent the time of the tectonic event provided that the related erosional response propagated linearly as a function of distance through the Alpine landscape. The c. 31.5 Ma age inferred from the intercept at origin is in remarkable agreement with the age for the slab breakoff, plutonic emplacement and enhanced exhumation in the back of the Alps (30–32 Ma)^10^,^11^,^12^,^13^,^14^. The erosional signal, also recorded in the Gonfolite Lombarda group at ~31Ma, is consistent with this picture (red dot on Fig. 5)^31^,^32^. This corroborates the idea that slab breakoff and related surface uplift 32–30 Ma ago (Fig. 1b) was the initial trigger, or principal driver, for the erosional signal recorded at delayed intervals in the different sections of the Molasse foreland basin. Accordingly, the general increasing trend in sediment flux (Fig. 2a) paired with continuous megafan progradation during the Late Oligocene (Fig. 2b) was accentuated by distinct pulses of sediment discharge. In the next section, we propose a mechanism that explains how these sediment flux pulses propagated through the system.

Rates of surface erosion by mountainous streams such as the Alps strongly depend on the stream gradients and the erodibility of the underlying bedrock^41^. For a given water discharge, fluvial erosion into bedrock tends to be fast for steeper channels and bedrock with higher erodibilities such as sandstones, mudstones and schists^41^, while dissection slows down when bedrock with low erodibilites such as granites, quartzites and gneisses^42^ become exposed to the surface^41^. In addition, in tectonically active landscapes such as the Alps between the Late Oligocene and the Miocene, channels steepen in response to fast rock uplift^43^, thereby promoting erosion and driving large sediment fluxes into the basin. We use these slope dependent incision mechanisms^44^ to explain the first immediate regional signal at c. 30 Ma, when slab breakoff and surface uplift in the back of the Alps promoted steeper slopes^18^, faster erosion and larger sediment fluxes into the Molasse basin^19^. The result was a first regional and immediate response in the basin, characterized by a switch from ‘flysch’-type basin underfill to ‘molasse’-type overfill paired with shifts to continental depositional environments in the foreland basin (1^st^ signal on Fig. 2b)^21^. Several My later, sediment pulses paired with larger grain sizes and provenance change to more crystalline material (red stars on Fig. 3), marking the arrival of a 2^nd^ signal (Fig. 2b), most likely occurred as the erosional front reached the crystalline core in the back of the Alps (Fig. 1b). The shape of the response then takes the form of a “whiplash”^27^ where sediment fluxes propagate as distinct waves into the foreland as suggested by Gasparini *et al*.^27^. These mechanisms thus occur as delayed responses to a long-term transience in surface erosion and landscape evolution, which are explained in the following paragraph.

**Figure 6:**
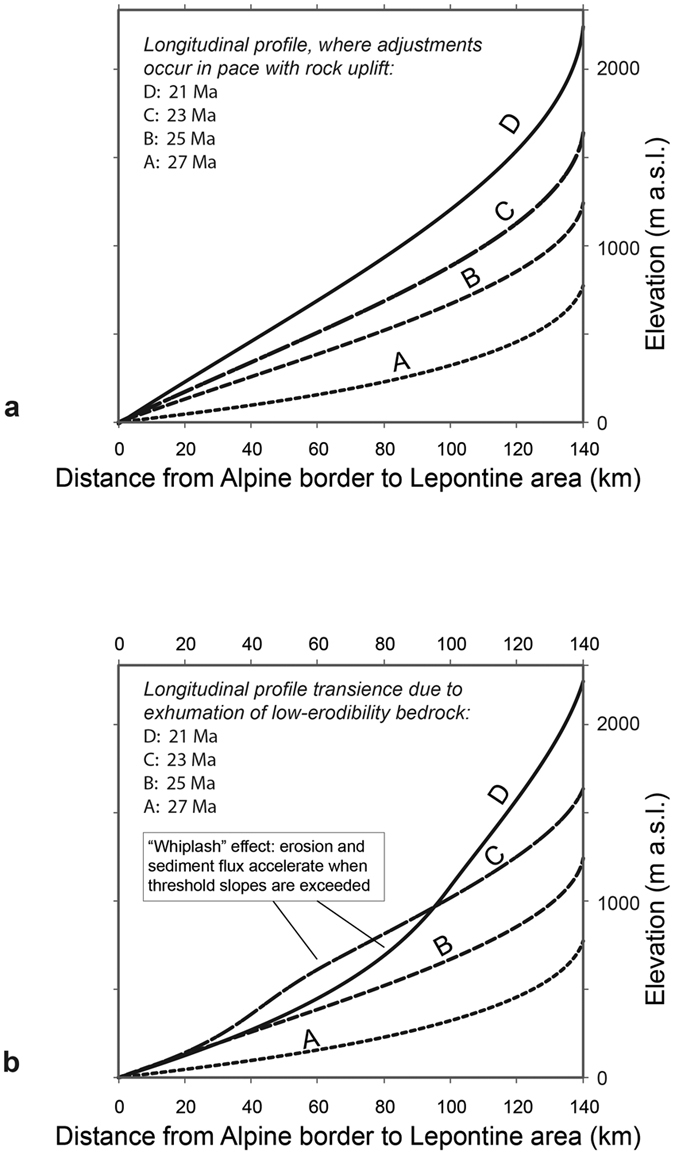
Hypothetic evolution of stream profiles in the Alps. (**a**) Steady adjustment of longitudinal streams to ongoing rock uplift. In case of zero perturbation, the gradient of a stream increases at each location, while the stream’s concavity remains constant. Steepening of the longitudinal stream profile continues until steady state conditions between rock uplift and surface erosion are reached (profile D). (**b**) ‘Whiplash’^27^ effect, exemplified for the longitudinal profile of the Hörnli stream between the drainage divide in the rear of the Alps and the point of entry in the foreland basin. Slab breakoff in the back of the Alps between 32–30 Ma resulted in continuous rock uplift. Streams responded by headward retreat and steepening of the stream gradients while maintaining a graded profile (situations A and B, same as Fig. 6a). As uplift proceeded and crystalline bedrock with larger erosional thresholds became exhumed, the streams adapted a transient convexity where these lithologies were exposed on the surface (situation C). Once streams had sufficiently steepened so that their stream power exceeded the larger erosional thresholds, the streams rapidly re-adapted graded longitudinal profiles (situation D) through downcutting into the convexity. The result was a secondary pulse of sediment into the foreland basin, associated with larger clasts and higher contributions of crystalline lithotypes (exhumation of crystalline bedrock) responsible for the convexity. The evolution of elevations through time has been extracted and modified from Schlunegger and Kissling^18^.

Channels respond to rock uplift through steepening their gradients^41^,^43^,^44^,^45^. If these adjustments occur in pace with increasing rock uplift rates, then channels maintain their concavities, but they will increase, at each location, their gradients (longitudinal stream profiles from A to D, Fig. 6a) until rock advection through uplift is fully compensated by fluvial incision (steady state)^41^,^43^,^44^,^45^. In such a scenario, sediment fluxes to the basin increase continuously and megafans steadily prograde into the basin until steady state conditions are reached. A perturbation to these processes is introduced by the progressive exposure of bedrock with lower erodibilities such as granites, gneisses and quartzites. These lithologies offer larger erosional thresholds^42^, thereby retarding surface response to rock uplift with the effect that longitudinal stream profiles take a transient convex shape (stream profile C on Fig. 6b), where bedrock with larger erosional thresholds is exposed to the surface. This transience is maintained until the landscape has sufficiently steepened so that stream power, which is the product between the channel’s slope and water discharge^45^, exceeds the erosional thresholds. At this point, the removal of the transient convexities and the re-adjustment to a graded longitudinal stream profile (evolution from C to D on Fig. 6b) induce a period of fast incision, thereby releasing large volumes of sediment into the streams^27^. Such a mechanism further promotes fluvial erosion through a positive feedback where larger bed load concentrations enhance fluvial dissection into bedrock^27^, thereby supplying large volumes of sediment with abundant crystalline clasts into the foreland basin. We use these mechanisms to explain the delayed arrival of the 2^nd^ signal in the basin.

If our hypothesis is correct, such a record of transient erosional response offers a natural laboratory for exploring sediment-flux dependent bedrock incision processes^27^, and thus allows a reduction of the range of erosion formulations needed in models of landscape evolution^46^. In addition, the dual stratigraphic record of a unique slab breakoff event, with a first immediate synchronous response at c. 30 Ma (1^st^ signal on Fig. 2b), and a second spatially diachronous signal 6–8 Ma later (2^nd^ signal on Fig. 2b), emphasizes the transient nature of landscape response to deep-seated tectonic processes over geological timescales. Our results thus indicate that the landscape’s response to a tectonic event can take several millions of years, as has also been suggested by the results of landscape evolution models^47^. This implies that the stratigraphic records in sedimentary basins to tectonic perturbations can be significantly protracted (several Ma) and possibly non-unique. This highlights the need for accurate chronological frameworks within entire source-to-sink systems where the scope is to invert stratigraphic records of large-scale tectonic processes.

## Methods

### Tectonic and chronostratigraphic framework

This paper is mainly based on a compilation of chrono-stratigraphic data from the Alps and the adjacent Swiss Molasse basin. The evolution of the Alps between the time of slab breakoff at 32–30 Ma and 20 Ma is taken from Schlunegger and Kissling (2015)^18^. These authors restored width, exhumation, and exposed bedrock for these times through balancing the shortening in the Alps^14^ and the foreland^7^,^34^,^40^, constrained by the cumulative 160-km-deep subduction of the European lithospheric mantle since 30 Ma^48^. The restorations of these authors also consider isostatic compensations of deep crustal loads related to the subducted slab, plus surface loads and buoyancy forces exerted by the stack of crustal material^18^. The results of these reconstructions revealed that the distance between the back of the Alps and the proximal basin border has remained nearly constant, at least between 30 and 20 Ma.

We take advantage of an extensive database from previous studies, which established a high-resolution chrono-stratigraphic framework through magneto-polarity stratigraphies combined with micro-mammal biostratigraphy at Necker^7^, Rigi^9^ and Thun^8^. These sections chronicle the evolution of the Hörnli, Rigi and Napf megafans, respectively. The uncertainties in the ages range between <0.5 and 1 Ma depending on the correlation of the magneto-polarity stratigraphies of individual sections with the magneto-polarity time scale. Sediment accumulation rates were calculated using non-decompacted stratigraphic thicknesses and the chronological records.

### Sedimentary features and palaeoenvironments

Clast size evolution as presented on Fig. 3 has been compiled from the literature for the Necker section^30^ representing the Hörnli megafan, and the Thun^28^ section where the Napf megafan deposits are exposed. New data was additionally collected for the sake of this paper for the Rigi deposits. Maximum clast sizes were measured with a meter stick in the field, where the mean of the five largest clasts per 4–5 m^2^ of outcrop was determined.

The sedimentological interpretation of the conglomerate suites is mainly based on the sedimentary fabric that has been analyzed in previous studies^7^,^24^,^28^, where the results are compiled in this paper. Prior to the inferred arrival of the signal, clast-supported and well-sorted conglomerates with shallow-inclined, m-scale cross-beds (<10°) with dips perpendicular to sole marks indicate a meandering/wandering pattern of the trunk channels where runoff was most likely perennial^28^. Upon arrival of the inferred signal, massive-bedded conglomerate beds with a clast-supported and well-sorted fabric, and disappearance of sole marks orthogonal to internal stratifications, were used to infer the occurrence of braided streams^28^. The conglomerate beds of the uppermost conglomerate-mudstone alternations, however, display a matrix-supported fabric in some conglomerate units with a moderate sorting. Some of these conglomerate beds also have a ribbon-shaped geometry. These arguments were used to infer the occurrence of torrential floods where streams had a local source situated in the Alpine border at that time^7^,^24^,^25^,^28^,^29^.

### Provenance analysis

The provenance of the conglomerates is mainly based on petrographic comparisons between clast types in the sections and preserved lithofacies in the hinterland. Several previous studies have documented that the sedimentary clasts at the Rigi^25^ and Necker sections (deposits of the Hörnli fan, Fig. 3)^7^,^26^,^30^ mainly comprise siliceous and micritic limestone clasts plus dolomite constituents. Related lithologies are currently encountered within the Helvetic and Penninic sedimentary nappes that make up the Alpine front, plus the Austroalpine nappes that form the orogenic lid^14^. However, ongoing metamorphosis at upper prehnit/pumpellyit and lower greenshist conditions of the Helvetic nappes until 20 Ma^14^ precludes the consideration of this litho-tectonic unit as potential material source. Also at Rigi and Necker, granitic clasts are non-metamorphosed and have preserved their original late Palaeozoic fabric, implying that they were most likely derived from the crystalline basement of the Austroalpine nappes situated in the back of the Alps^7^,^25^,^26^,^30^. We thus interpret the succession of clast types starting from sedimentary lithotypes and closing with crystalline constituents to reflect a normal unroofing sequence where erosion successively reached deeper crustal levels. Similarly, studies of the Thun petrography^29^ (section that chronicles the evolution of the Napf fan) have shown that the clast types include siliceous and micritic limestones derived from the Penninic and possibly Austroalpine sedimentary nappes. The subsequent arrival of quartzite clasts with a greenshist fabric and abundance of epidote heavy minerals suggests an origin situated in the Saas-Zermatt ophiolites^23^ between the Austroalpine and the Penninic nappes (Fig. 1b), and in the crystalline core of the Penninic nappes (quartzite clasts). This sequence of material arrival thus reflects a normal unroofing sequence where erosion has cut into successively deeper levels in the back of the Alps. Flysch sandstone clasts, that form the predominant clast type at the top of all sections (Fig. 3), have been derived from north Penninic flysch nappes mainly because of abundant apatite heavy minerals and the absence of spinel constituents that characterized the northern Penninic sedimentary realm^49^,^50^. Related nappes are currently found at the front of the Alps (e.g., Schlieren-Flysch).

We infer that the drainage divide between the streams draining to the North and the South was situated in the region of the Cressim-Vanzone-Mischabel-backfold c. 10–20 km north of the Insubric Line^14^ (Fig. 1b). This is based on: (1) the occurrence of boulders from the Bergell batholith in the Gonfolite Lombarda conglomerates^32^ but not in the Molasse Basin, and (2) abundant epidote heavy minerals, derived from the ophiolitic bedrock, that were encountered in the Molasse^29^ but not in the Gonfolite Lombarda group. The Bergell unit straddles this backfold, while widespread exposures of ophiolites (Saas-Zermatt zone, Malenco unit) occur north of it^14^. Accordingly, there is strong evidence that the topographic rise formed through this backfold served as major drainage divide at least between 30 and 20 Ma^14^,^15^.

### Palaeoclimate proxy records

We use global deep-sea oxygen and carbon isotope records from benthic foraminifera that were compiled from more than 40 drilling sites around the globe by Zachos *et al*.^36^ as proxy for palaeoclimate records. At a more local scale, stable isotope measurements were accomplished on charophytes embedded in lacustrine limestones^38^, and caliche nodules in palaeosoils^39^. We adapted both the datasets and the interpretation of the corresponding authors in this paper. The shift towards heavier carbon and lighter oxygen isotopes in the caliche nodules was used to infer a change towards a more continental and warmer palaeoclimate^38^, where the plant coverage was less dense^37^,^38^,^39^. Likewise, shifts towards lighter oxygen isotopes in charophytes also suggest that conditions became more continental^38^. This was considered as consistent with the changes in the palaeofloral records^37^,^38^ that are characterized by the disappearance of palms, walnuts (Engelhardia) and members of the cypress family (Taxodiacea), and the new-appearance of pines (Pinaceae), legumes (Leguminosae) and poplars (Populoids).

## Additional Information

**How to cite this article**: Schlunegger, F. and Castelltort, S. Immediate and delayed signal of slab breakoff in Oligo/Miocene Molasse deposits from the European Alps. *Sci. Rep*. **6**, 31010; doi: 10.1038/srep31010 (2016).
